# Cyanidin-3-rutinoside attenuates methylglyoxal-induced protein glycation and DNA damage via carbonyl trapping ability and scavenging reactive oxygen species

**DOI:** 10.1186/s12906-016-1133-x

**Published:** 2016-05-23

**Authors:** Thavaree Thilavech, Sathaporn Ngamukote, Damien Belobrajdic, Mahinda Abeywardena, Sirichai Adisakwattana

**Affiliations:** Program in Biomedical Sciences, Graduate School, Chulalongkorn University, Bangkok, 10330 Thailand; Department of Nutrition and Dietetics, Faculty of Allied Health Sciences, Chulalongkorn University, Bangkok, 10330 Thailand; CSIRO Food and Nutrition, Adelaide, SA 5000 Australia

**Keywords:** Cyanidin-3-rutinoside, Methylglyoxal, Protein glycation, Oxidative DNA damage

## Abstract

**Background:**

Advanced glycation end-products (AGEs) play a significant role in the development and progression of vascular complication in diabetes. Anthocyanin has been recently reported to possess antiglycating activity. This study aimed to determine whether a naturally occurring anthocyanin, cyanidin-3-rutinoside (C3R) inhibits methylglyoxal (MG) induced protein glycation and oxidative protein and DNA damage.

**Methods:**

C3R (0.125–1 mM) was incubated with bovine serum albumin and MG (1 mM) for 2 weeks. The formation of fluorescent AGEs was measured by using spectrofluorometer and thiol group content were used to detect protein oxidative damage. Gel electrophoresis was used to determine whether C3R (0.125–1 mM) reduced DNA strand breakage in a glycation model comprising lysine, MG and/or Cu^2+^. The generation of superoxide anions and hydroxyl radicals were detected by the cytochrome *c* reduction assay and the thiobarbituric acid reactive substances assay. MG-trapping capacity was assessed by high performance liquid chromatography (HPLC).

**Results:**

C3R (0.25–1 mM) reduced the formation of fluorescent AGEs and depleted protein thiol groups in bovine serum albumin mediated by MG. At 1 mM C3R inhibited oxidative DNA damage in the glycation model (*p <* 0.05) and at 0.5–1 mM prevented Cu^2+^ induced DNA strand breakage in the presence of lysine and MG. The findings showed that C3R reduced the formation of superoxide anion and hydroxyl radicals during the glycation reaction of MG with lysine. C3R directly trapped MG in a concentration and time dependent manner (both *p <* 0.001).

**Conclusions:**

These findings suggest that C3R protects against MG-induced protein glycation and oxidative damage to protein and DNA by scavenging free radicals and trapping MG.

## Background

Methylglyoxal (MG) is recognized as the most reactive glycating agent, generated during glycation and endogenously via carbohydrate, lipid and protein metabolism, especially during the glycolysis pathway [1, 2]. Whilst the glyoxalase defense system converts the damaging MG into D-lactate via the glyoxalase enzyme complex, elevated tissue and plasma levels of MG are commonly observed in diabetes [3, 4]. Methylglyoxal hydroimidazolone (MG-H1) is reported to be the most abundant MG-derived AGE modification in vivo leading to protein dysfunction [5]. The interaction of AGEs with a receptor for AGEs (RAGE) triggers signal transduction by activation of the MAPK pathway, resulting in reactive oxygen species (ROS) overproduction and inflammation [6]. The glycation reaction of amino acids with MG directly generates ROS, causing damage to cellular protein and DNA [7–10]. In particular, oxidative damage of DNA is associated with the development of several pathologies, including physiological ageing, metabolic syndrome, diabetes, cancer and cardiovascular diseases [11–13].

MG has been recognized as a potential target for intervention and novel pharmacological strategies are being developed to limit its accumulation and minimize its detrimental effects. Of these strategies, the ability for some AGE inhibitors to trap MG has received considerable interest [14]. The early clinical trials with aminoguanidine (AG) showed great promise as an AGEs inhibitor. However, studies in people with diabetic nephropathy were terminated following safety and efficacy concerns [15, 16]. Alternatively, studies on AGE inhibitors from natural products show more promise to combat AGE-associated diseases by scavenging free radicals, or by directly trapping MG. In this regard, pyridoxamine, a form of naturally occurring vitamin B_6_ and inducer of glyoxalase enzyme expression -isothiocyanates and sulforaphanes found in cruciferous vegetables, have been reported to have some promise [17, 18]. In particular, anthocyanins, the colourful pigment in various fruits and vegetables reduced ROS generation in human HepG2 cells exposed to a high glucose environment [19]. This could explain, in part their biological effectiveness as anti-oxidant, anti-carcinogenic, anti-microbial and anti-inflammatory agents and their role in ameliorating hyperglycemia by improving insulin sensitivity via the cAMP-activated protein kinase pathway in diabetic mice [20–23]. A derivative of anthocyanin, cyanidin-3-rutinoside (C3R), maybe particularly important as it delays postprandial glycemia by inhibiting α-glucosidase and pancreatic α-amylase which play an important role in glucose metabolism [24–26]. Most recently, C3R inhibited ribose-, fructose-, glucose- and galactose-induced protein glycation and oxidation in vitro [27]. Collectively these findings suggest that C3R may prevent MG-induced AGEs formation and oxidative protein and DNA damage which have not been investigated previously.

This study aimed to determine whether C3R inhibited MG-induced protein glycation (formation of AGEs) and oxidation (depletion of thiol groups) in vitro, and reduced MG/lysine-induced DNA damage using a plasmid DNA assay. To investigate possible mechanisms of action, we evaluated the role of C3R in reducing the generation of superoxide anion and hydroxyl radicals as well as direct scavenging of the MG. Due to the complexity of the glycation cascade, in vitro models were used as they enabled identification of the pathways of action.

## Methods

### Chemicals and Reagents

Bovine serum albumin (BSA) fraction V was purchased from Fisher scientific (Hudson, NH, USA). 40 % solution methylglyoxal (MG), 5,5′-dithiobis (2-nitrobenzoic acid) (DTNB), *o*-phenylenediamine (*o*-PD), 5-methylquinoxaline (5-MQ), 2-deoxy-D-ribose, 2-thiobarbituric acid (TBA), L-cysteine and aminoguanidine hydrochloride (AG) were purchased from Sigma (St. Louis, MO, USA). L-lysine hydrochloride was purchased from Himedia (L.B.S. Marg, MB, India). Trichloroacetic acid, methanol (gradient grade for liquid chromatography) and guanidine hydrochloride were purchased from Merck (Darmstadt, Germany). Cytochrome *c* was obtained from Affymetrix (Santa Clara, CA, USA). Cyanidin-3-rutinoside chloride was synthesized from quercetin-3-rutinoside according to a previous study [28].

### Bovine serum albumin (BSA)-methylglyoxal assay

The glycated BSA was generated according to a previous study [29]. In brief, 10 mg/mL of BSA was incubated with 1 mM MG in 0.1 M phosphate buffered-saline (PBS, pH 7.4) containing 0.02 % sodium azide in the presence or absence of C3R (0.125–1 mM) or AG (1 mM) at 37 °C for 1 and 2 weeks. PBS was used as a blank and dimethylsulfoxide (DMSO) at a final concentration 4 % was used as solvent to dissolve C3R. The formation of fluorescent AGEs was determined using a spectrofluorometer (Perkin Elmer®, Finland) at excitation wavelength of 355 nm and emission wavelength of 460 nm. The results were expressed as percentage of inhibition:

$$ \mathrm{Inhibition}\ \mathrm{of}\ \mathrm{fluorescent}\ \mathrm{AGEs}\left(\%\right)=\left[\left(\left({\mathrm{F}}_{\mathrm{C}}\hbox{-} {\mathrm{F}}_{\mathrm{C}\mathrm{B}}\right)\hbox{-} \left({\mathrm{F}}_{\mathrm{S}}\hbox{-} {\mathrm{F}}_{\mathrm{S}\mathrm{B}}\right)/\left({\mathrm{F}}_{\mathrm{C}}\hbox{-} {\mathrm{F}}_{\mathrm{C}\mathrm{B}}\right)\right)\right]\times 100 $$

F_C_ was fluorescence intensity of MG with BSA and F_CB_ were the fluorescence intensity of blank. F_S_ and F_SB_ were the fluorescence intensity of C3R or AG with BSA/MG and blank, respectively.

### Determination of thiol group content

Thiol groups in glycated BSA were measured after 1 and 2 weeks of incubation according to the Ellman assay [30]. Briefly, BSA samples (10 μL) were mixed with 2.5 mM DTNB (90 μL) for 15 min. The absorbance was measured at 412 nm. The free thiol concentration was calculated by using a standard curve of L-cysteine.

### Analysis of DNA strand breaks

The cleavage of plasmid DNA was analyzed according to a previous report [9]. Briefly, pUC19 plasmid DNA was purified from Escherichia coli (E. coli) cultures using a QIAprep®spin miniprep kit (Santa Clarita, USA). Plasmid DNA (250 ng) was incubated with 50 mM lysine, 50 mM MG, and 0.125–1 mM C3R with or without 300 μM Cu^2+^ at 37 °C for 3 h. Samples were frozen immediately at −20 °C to stop reactions. After 90 min, the plasmid DNA was mixed with DNA loading dye and then resolved by 0.8 % agarose gel electrophoresis at 80 V in TBE buffer for 60 min. Plasmid DNA fragments were visualized and photographed by a Gel Doc imager (Syngene, UK). The relative amounts of supercoiled (SC) and open circular (OC) DNA was quantified by the intensities of the band obtained using GeneTools software (Syngene, UK) and the percentage of opened circular DNA (% OC) was calculated using the following equation. The results were expressed as relative % OC after subtracting by %OC of control (DNA alone).

$$ \%\;\mathrm{O}\mathrm{C}=\frac{\mathrm{Intensity}\;\mathrm{of}\;\mathrm{O}\mathrm{C}}{\mathrm{Intensity}\;\mathrm{of}\;\mathrm{O}\mathrm{C}+\mathrm{S}\mathrm{C}}\times 100 $$

### Determination of superoxide anions

The level of superoxide anions was determined by measurement of cytochrome *c* reduction assay according to a previous method [9]. In brief, 10 mM lysine and 10 mM MG was incubated in the presence or absence of C3R at concentration 0.125–1 mM. The reduction rate of cyctochrome *c* was measured at room temperature using a spectrophotometer at 550 nm at 10 min intervals for 50 min. The level of reduced cytochrome *c* was calculated based on the extinction coefficient for cytochrome *c* (ε = 27,700 M^−1^cm^−1^).

### Determination of hydroxyl radicals

Thiobarbituric acid reactive substances (TBARS) assay was used to evaluate the level of hydroxyl radicals according to a previously described method [31]. Briefly, the reaction mixture (0.2 mL total volume) containing 10 mM lysine, 10 mM MG, and 20 mM 2-deoxy-d-ribose was incubated at 37 °C with or without C3R (0.125–1 mM). After 3 h, 0.2 mL PBS and 0.2 mL TCA (2.8 % w/v) was added to the reaction mixture, followed by 0.2 mL thiobarbituric acid (TBA). The solution was boiled at 100 °C for 10 min and then cooled to room temperature. The degradation of 2-deoxy-d-ribose was measured at a wavelength 532 nm using a spectrophotometer. Hydroxyl radicals were expressed as the level of TBARS which was quantified by using standard curve of malondialdehyde.

### Determination of MG-trapping ability

C3R was incubated with MG at various molar ratios including 0.25:1, 0.5:1, 1:1, 2:1, 4:1 and 16:1 in 0.1 M PBS, pH 7.4. AG was used as positive control to incubate with MG at 1:1 molar ratio. The reaction mixtures were incubated at 37 °C for 1 and 24 h. After the incubation, 20 mM *o*-phenylenediamine (*o*-PD) was added to stop the reaction by converting the remaining MG into 2-methylquinoxaline (2-MQ). The quantification of MG was based on the determination of its derivative compound (2-MQ) using HPLC [32]. An Inersil-ODS3V C_18_ column (150 × 4.6 mm i.d.; 5-μm particle size) was used along with a LC-10 AD pump, SPD-10A UV-vis detector and LC-Solution software (Shimadzu Corp., Kyoto, Japan). The absorbance was recorded at 315 nm and the injection volume was 10 μL. An isocratic program was performed with 50 % HPLC grade water and 50 % methanol (v/v) with a constant flow rate set at 1 mL/min. The total running time was 14 min and the internal standard was 0.06 % (v/v) 5-methylquinoxaline (5-MQ) in methanol. The percentage of MG reduction was calculated using the equation below:

$$ \%\mathrm{M}\mathrm{G}\;\mathrm{trapping}\;\mathrm{ability}=\frac{\mathrm{Amount}\;\mathrm{of}\left(\mathrm{M}\mathrm{G}\;\mathrm{in}\;\mathrm{control}\hbox{-} \mathrm{M}\mathrm{G}\;\mathrm{in}\;\mathrm{C}3\mathrm{R}\right)}{\mathrm{Amount}\;\mathrm{of}\;\mathrm{M}\mathrm{G}\;\mathrm{in}\;\mathrm{control}}\times 100 $$

### Statistical analysis

Data are presented as the arithmetic means ± SEM for each treatment group (*n* = 3). The effect of C3R on the formation of AGEs, protein oxidation, DNA strand break and MG-induced generation of hydroxyl radical was analyzed by one-way ANOVA. The effect of C3R on MG-induced generation of superoxide anion was determined by two-way repeated measures ANOVA. The effect of C3R on MG-trapping ability was determined by a two-way ANOVA. Differences between treatments were analyzed by a Tukey post hoc test. These analyses were performed using SPSS Statistics 17.0 (SPSS Inc., Chicago, IL, USA). A P value of less than 0.05 was taken as the criterion of significance.

## Results

### C3R and the formation of AGEs

**Fig. 1:**
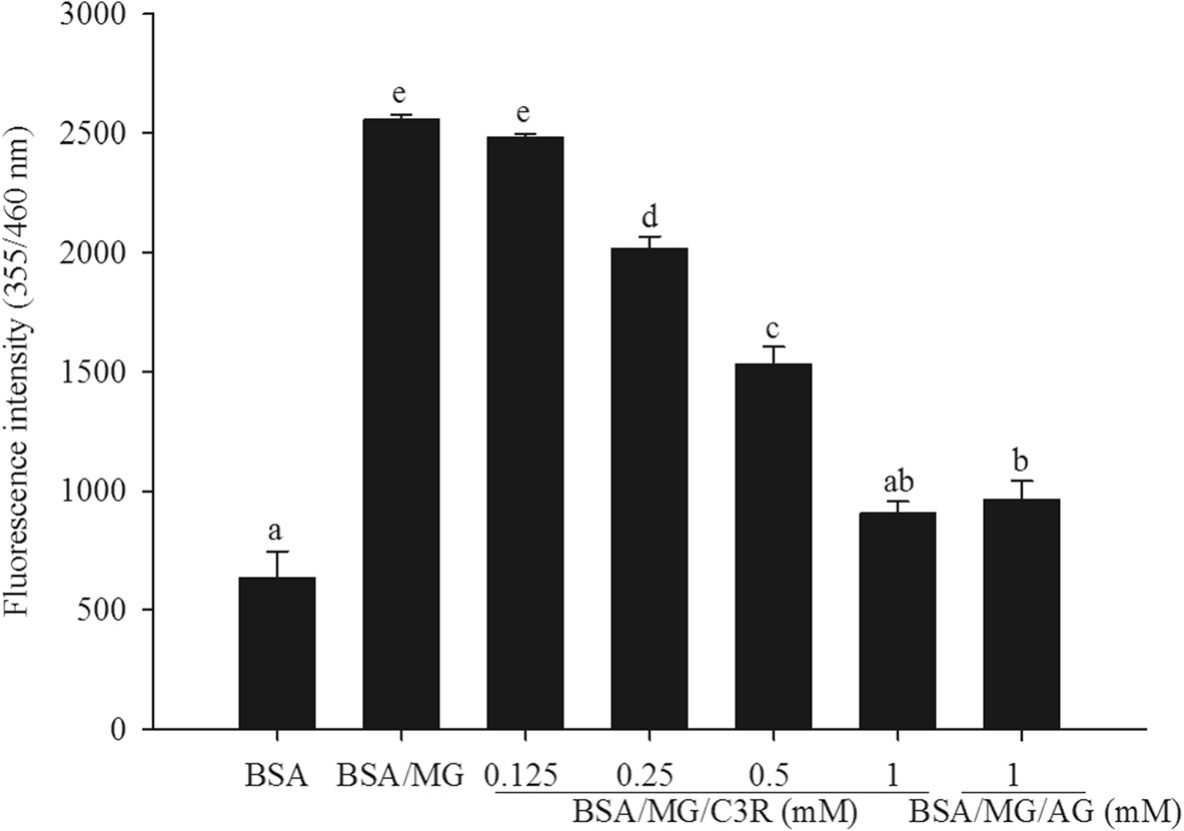
The effects of cyanidin-3-rutinoside (C3R) and aminoguanidine (AG) modulating the formation of fluorescent glycated protein in the BSA/methylglyoxal (MG) assay. The results are presented as mean ± SEM (*n* = 3). Significance is shown in groups that do not share a common letter (*p* < 0.05)

The addition of MG to BSA in the BSA/MG assay caused a 5-fold increase in the formation of fluorescent AGEs (Fig. 1). This MG induced glycation of BSA was reduced when C3R was added at a level of 0.25 mM or above (*p* < 0.05) and the reduction was greatest when C3R was added at 1 mM (65 %) such that glycation levels were similar to when no MG was present. The addition of AG as a positive control inhibited the formation of fluorescent AGEs by a similar amount as 1 mM C3R.

### C3R and protein oxidation

**Fig. 2:**
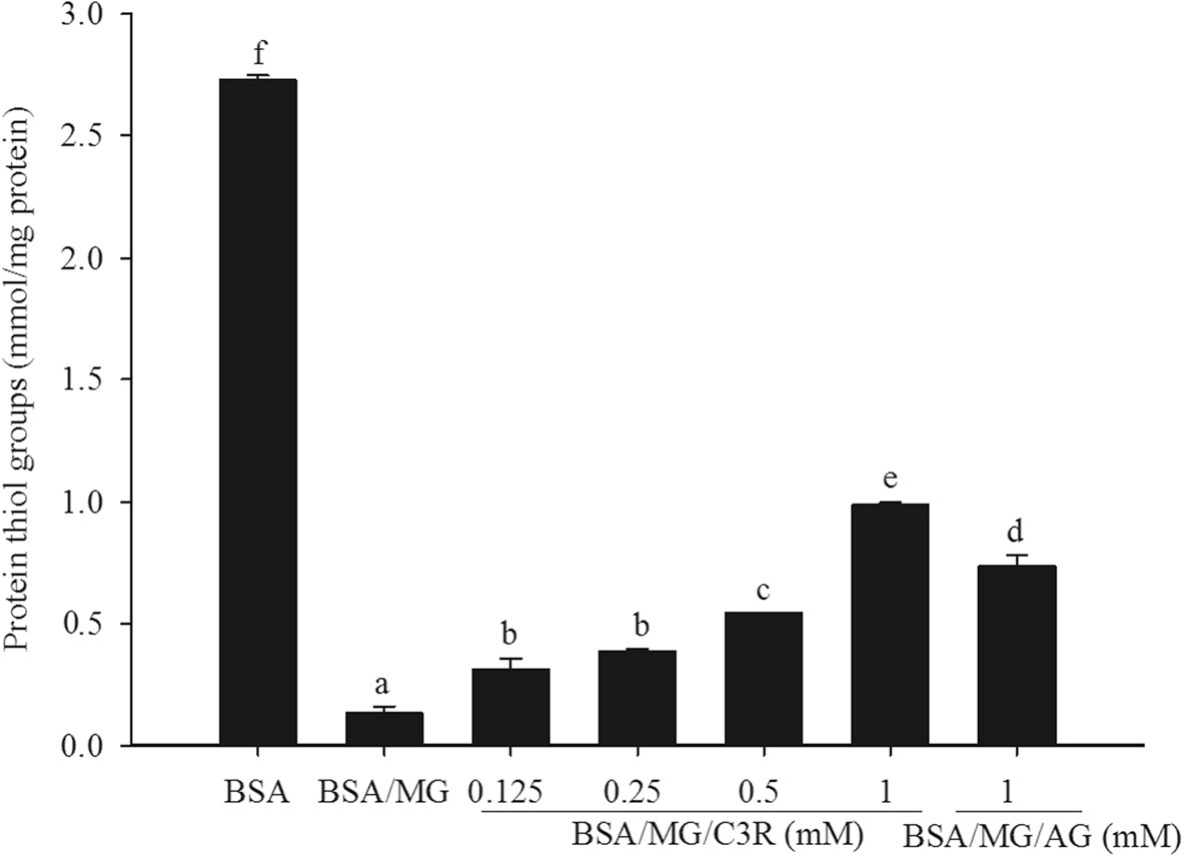
The effects of cyanidin-3-rutinoside (C3R) and aminoguanidine (AG) modulating the level of protein thiol groups in the BSA/methylglyoxal (MG) assay. The results are presented as mean ± SEM (*n* = 3). Significance is shown in groups that do not share a common letter (*p* < 0.05)

Incubation of BSA with MG dramatically reduced the number of thiol groups by 95 %. This reduction in thiol groups was impaired by C3R at concentrations of 0.125 mM or greater (Fig. 2). At the highest concentration of C3R (1 mM) tested, there were 33 % more thiol groups than BSA/MG (*p* < 0.05), an effect that was greater than that seen when BSA/MG was incubated with AG (1 mM).

### C3R and MG-induced DNA strand breakage

**Fig. 3:**
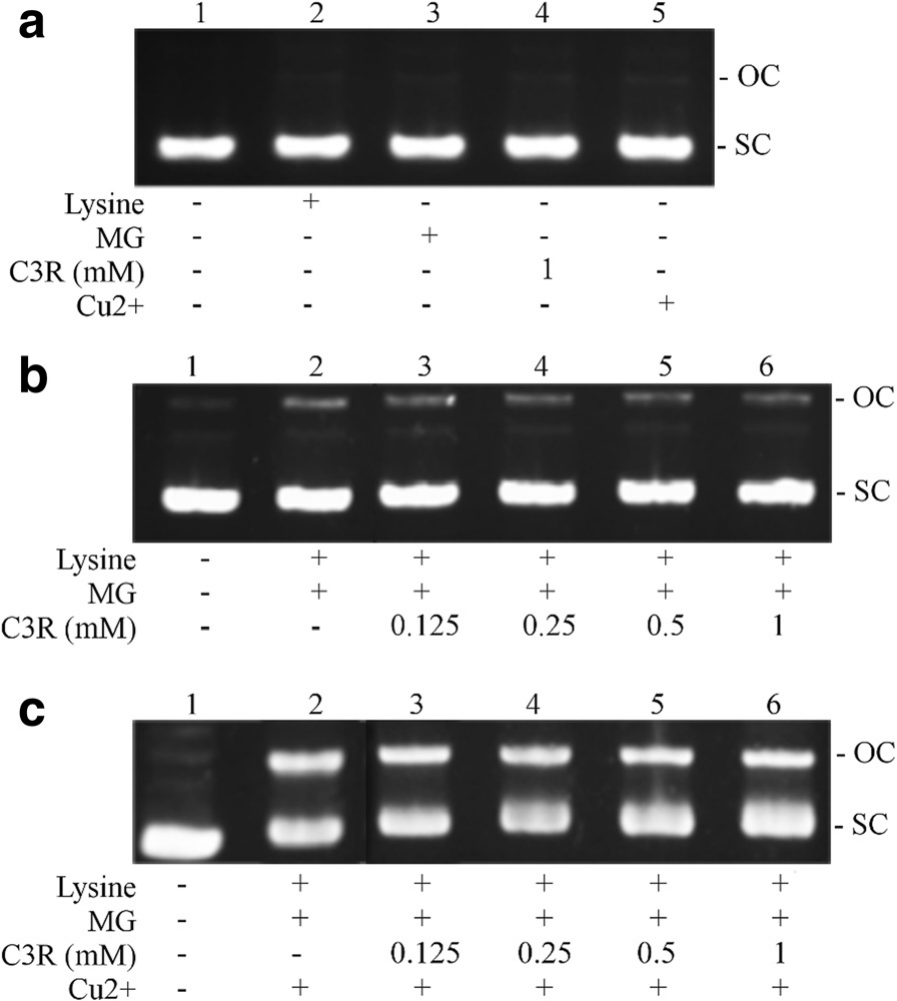
The effects of cyanidin-3-rutinoside (C3R) on DNA cleavage-mediated by glycation of lysine with methylglyoxal (MG) in the absence or presence of Cu^2+^. The major band corresponds to supercoiled form (SC), and damaged plasmid DNA is represented as opened circular form (OC). pUC19 DNA (0.25 μg) was incubated with the following: **a** Lane 1, DNA alone; Lane 2, 50 mM lysine; Lane 3, 50 mM MG; Lane 4, 300 μM CuSO_4_; Lane 5, 1 mM C3R. **b** Lane 1, DNA alone; Lane 2, 50 mM lysine + 50 mM MG; Lane 3, 50 mM lysine + 50 mM MG + 0.125 mM C3R; Lane 4, 50 mM lysine + 50 mM MG + 0.25 mM C3R; Lane 5, 50 mM lysine + 50 mM MG + 0.5 mM C3R; Lane 6, 50 mM lysine + 50 mM MG + 1 mM C3R. **c** Lane 1, DNA alone; Lane 2, 50 mM lysine + 50 mM MG + 300 μM Cu^2+^; Lane 3, 50 mM lysine + 50 mM MG + 300 μM Cu^2+^ + 0.125 mM C3R; Lane 4, 50 mM lysine + 50 mM MG + 300 μM Cu^2+^ + 0.25 mM C3R; Lane 5, 50 mM lysine + 50 mM MG + 300 μM Cu^2+^ + 0.5 mM C3R; Lane 6, 50 mM lysine + 50 mM MG + 300 μM Cu^2+^ + 1 mM C3R

**Fig. 4:**
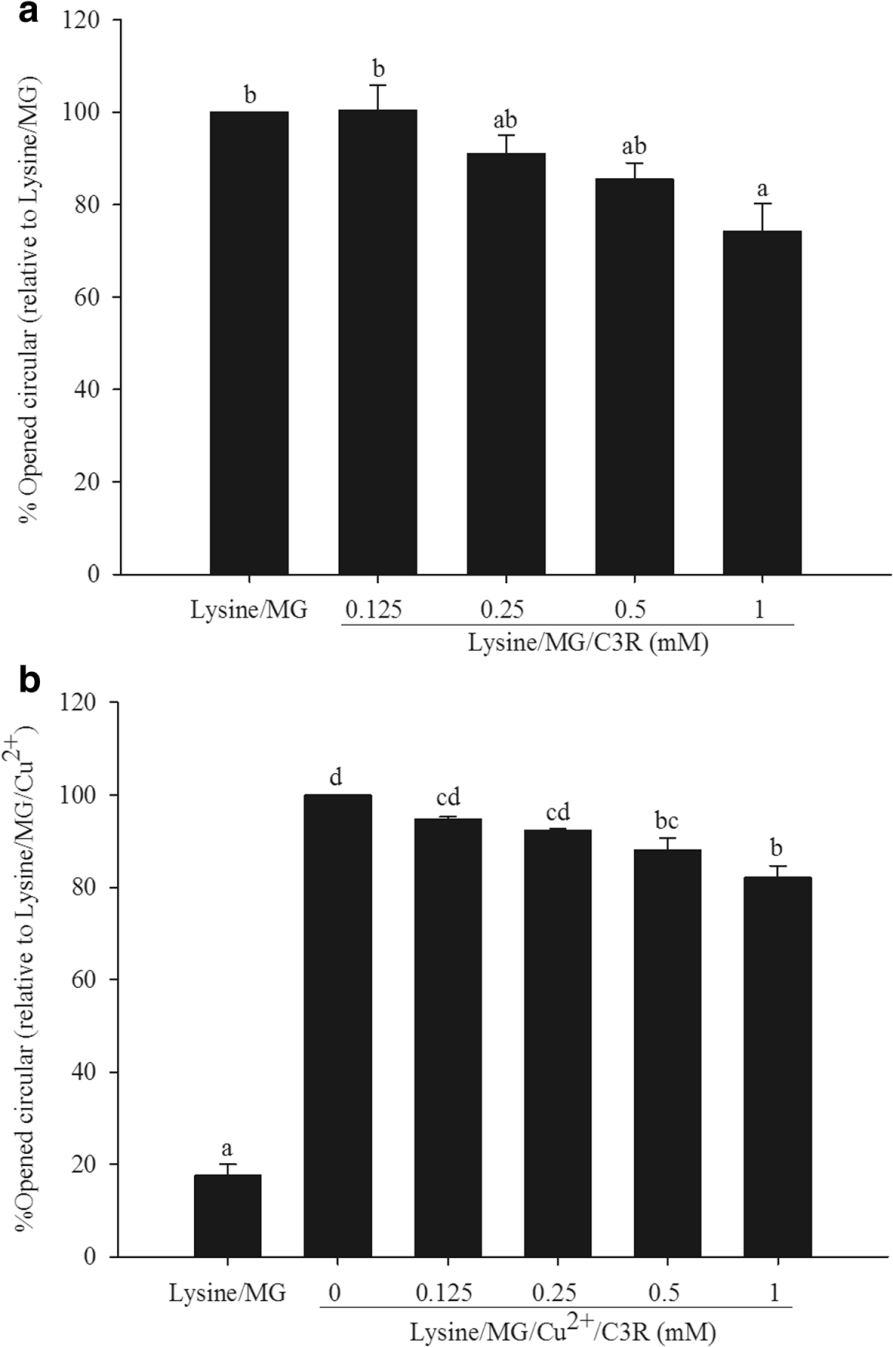
The effect of cyanidin-3-rutinoside (C3R) modulating % open circular (OC) form of plasmid DNA in lysine/methylglyoxal (MG)-induced DNA strand breakage in the absence (**a**) and presence of Cu^2+^ (**b**). The results are presented as mean ± SEM (*n* = 3). Significance is shown in groups that do not share a common letter (*p* < 0.05)

The addition of lysine, MG, Cu^2+^ or C3R to plasmid DNA did not cause DNA cleavage, as plasmid DNA remained in the supercoiled (SC) form (Fig. 3a). The addition of MG and lysine to plasmid DNA increased strand breakage of plasmid DNA by 69 % by increasing intensity of the open circular (OC) band (Fig. 3b) and was reduced by 26 % at the highest dose of C3R (1 mM) (Fig. 4a). In the presence of Cu^2+^, the cleavage of plasmid DNA was 77 % higher than lysine/MG without Cu^2+^ (Fig. 3c) and was markedly reduced by 12 % and 18 % at 0.5 and 1 mM of C3R, respectively (Fig. 4b).

### C3R and MG-induced generation of superoxide anion and hydroxyl radicals

**Fig. 5:**
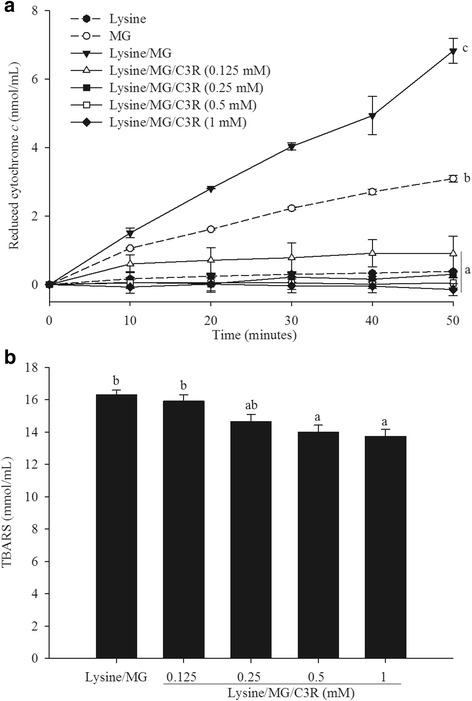
The effects of cyanidin-3-rutinoside (C3R) modulating the production of superoxide anion (**a**) and hydroxyl radicals (**b**) in lysine/methylglyoxal (MG)-induced glycation. The results are presented as mean ± SEM (*n* = 3). Significance is shown in groups that do not share a common letter (*p* < 0.05)

MG increased the formation of superoxide anion formation (as measured by reduced cytochrome c) over the 50 min incubation period which was significantly higher when MG was incubated with lysine (*p* < 0.001). The elevation of superoxide anion production caused by lysine/MG was prevented by all concentrations of C3R (Fig. 5a).

The lysine/MG induced generation of hydroxyl radicals (as measured by TBARS) was reduced when C3R was added at the level of 0.5 mM or above (Fig. 5b). The highest concentration of C3R (1 mM) resulted in a 20 % inhibition of hydroxyl radical generation.

### C3R and MG trapping ability

An evaluation of direct MG-trapping capacity was carried out in order to investigate whether C3R could directly scavenge MG. The percentage of MG-trapping efficiency of C3R was consistent with the increased concentration of C3R and time of incubation (*p* < 0.001). The increase in incubation time from 1 to 24 h increased efficiency of C3R to scavenge MG by 2-fold. In addition, the MG trapping ability of C3R was increased as the molar ratio of C3R to MG increased. The equal molar ratio of C3R to MG resulted in 21 % and 45 % MG-trapping capacity after 1 and 24 h of incubation. AG showed a higher trapping capacity compared to C3R as it trapped 90 and 95 % of MG when incubated with MG (1:1 molar ratio) for 1 and 24 h, respectively (Table 1).

**Table 1:** The percentage of methylglyoxal (MG)-trapping ability of cyanidin-3-rutinoside (C3R) and aminoguanidine (AG)

Time (h)	Molar ratio of C3R:MG						Molar ration of AG:MG
	0.25:1	0.5:1	1:1	2:1	4:1	16:1	1:1
1	5 ± 0.3	12 ± 1.3	21 ± 2.6	32 ± 2.0	42 ± 2.5	61 ± 1.9	90 ± 3.2
24	9 ± 1.2	21 ± 1.0	45 ± 0.7	57 ± 3.0	73 ± 3.2	89 ± 1.1	95 ± 4.6

The results are presented as mean ± SEM (*n* = 3)

## Discussion

The development of macrovascular and microvascular diabetic complications is associated with the formation and accumulation of advanced glycation end products (AGEs) [33, 34]. There is an important need to identify approaches that may prevent their formation [35, 36] and this study shows for the first time that cyanidin-3-rutinoside (C3R) effectively inhibited dicarbonyl intermediate methylglyoxal (MG)-derived AGEs formation at an immediate stage of the glycation process. C3R finally suppressed the formation of fluorescent AGEs and non-fluorescent AGEs in advanced stage of glycation process. This inhibitory activity of C3R on protein glycation was just as effective as aminoguanidine (AG) when highest dose (1 mM) was provided. These findings extend previous research which showed that C3R inhibited ribose-, fructose-, glucose- and galactose-induced AGEs formation during the initial stage of glycation associated with the reduction of Amadori product fructosamine [27]. Furthermore, berry and grape extracts containing anthocyanin also inhibited the formation of AGEs in an in vitro model involving fructose, MG and BSA [37]. It is likely that these protein glycation protective effects of the fruit extracts may be largely due to C3R content as it is the predominant anthocyanin component [38, 39]. These findings, taken together, suggest that C3R potentially impairs initiation and intermediate stages of protein glycation and supports the need for efficacy and bioavailability studies of C3R in animals and humans.

The effectiveness of C3R in reducing the formation of AGEs can be explained in part by the effect of C3R on scavenging superoxide anion and hydroxyl radicals. In the current study, we showed that in the presence of lysine and MG, C3R reduced the level of TBARS formation at concentrations of 0.25 mM or greater and maintained the reduced form of cytochrome *c* at background levels. C3R also prevented the depletion of thiol, a marker of protein oxidation with higher potency than AG when provided at an equal concentration (1 mM) in BSA/MG (1 mM) system. In previous report, the ROS production during glycation process in BSA/MG system showed potent to induce DNA strand breakage when used the higher concentration of MG (20 mM) for 10 and 21 days of incubation [40]. Protein cross-linking mediated by MG generates free radicals during this reaction including the MG-radical anion and cross-linked radical cation (MG-protonated cation) [41]. The MG-protonated cation is a precursor of fluorescent AGEs while MG-radical anions could donate an electron to oxygen molecule to generate a superoxide anion and hydroxyl radical [9, 10, 42]. The presence of a transition metal ion copper could stimulate the Fenton-like reaction to produce more highly reactive hydroxyl radicals that play an important role in mechanism of oxidative DNA strand breakage [11]. The ability of phytochemical compounds on the prevention of MG-induced protein glycation and DNA damage related to free radical scavenging activity has been reported [31, 42, 43]. Anthocyanin-rich extract containing high concentration of C3R also exhibits potent antioxidant activity [44, 45] and it is suggested that C3R may act as free radical scavenger to prevent oxidative damage of protein and DNA. These studies support previous reports that C3R inhibited the production of a secondary product of oxidation malonaldehyde from the degradation of 2-deoxyribose unit in DNA mediated by Fenton’s reagent as well as decreased ROS production and DNA damage in hydrogen peroxide-simulated RAW 264.7 murine macrophage cell [46, 47].

The current study demonstrated that C3R was able to directly trap MG which explains, in part its action in preventing the formation of AGEs and oxidative protein and DNA damage. The percentage of MG-trapping ability increased in a concentration dependent manner and these findings are supported by a previous study which showed that a purified C3R from blackcurrant extract trapped nearly 50 % of MG when it was incubated at a 1:1 ratio. The C3R-mono-MG adduct was identified as product from the reaction by using liquid chromatography electrospray ionization mass spectrometer (LC-ESI-MS) [48]. The molecular weight difference between the adduct (667 m/z) and the original C3R (595 m/z) is 72 which is same as the molecular weight of one molecule of MG. It has been reported the addition of MG on anthocyanin may probably form tautomers with transformation of carbonyl group of MG to hydroxyl group [49]. In the previous study, the MG addition reaction reacted with phenolic compounds at carbon atom with negative electron charge more than −0.24. Therefore, carbon number 2, 6, 7 and 15 of C3R is the possible location for MG addition reaction [48]. Chen et al. demonstrated the C3R/MG reaction for only 1 h [48] but the different molar ratio and incubation time between C3R and MG was evaluated in the current study. The reaction rate between C3R and MG at 1:1 molar ratio was nearly 2-fold higher when the incubation time was increased from 1 to 24 h. It suggests that the trapping ability of C3R is dependent on the concentration and time of incubation. In addition, flavonoids have also been shown to inhibit AGEs formation via MG-trapping ability [37, 50–53] which may be due to their chemical structure that consists of phenyl ring (A- and B-ring) and heterocyclic ring (C-ring). The major sites to conjugate with MG are the carbon positions at 6 and 8 on the A-ring [51] which is the same structure present in C3R and may explain their effectiveness in trapping MG [48].

## Conclusion

**Fig. 6:**
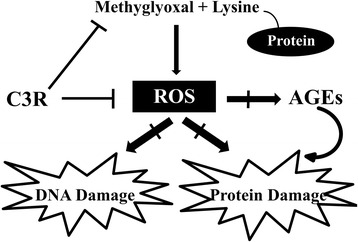
A schematic illustration of the effect of cyanidin-3-rutinoside (C3R) on the formation of methylglyoxal (MG)-induced protein glycation and oxidative protein and DNA damage. The scavenging of reactive oxygen species (ROS) and directly trapping of MG may be the major mechanisms of C3R to protect against protein and DNA damage

C3R inhibited MG-induced protein glycation and oxidation-dependent damage to bovine serum albumin and prevented lysine/MG-induced oxidative DNA damage. The inhibitory effect of C3R was attributed in part to its ability to scavenge ROS and directly trap reactive dicarbonyl MG (Fig. 6). These observations suggest that C3R may have anti-glycation potential for preventing diabetic complications.

## Abbreviations

2-MQ, 2-methylquinoxaline; AG, Aminoguanidine; AGEs, Advanced glycation end products; BSA, Bovine serum albumin; C3R, Cyanidin-3-rutinoside; MG, Methylglyoxal; ROS, Reactive oxygen species; TBAR, Thiobarbituric acid reactive substances.
